# Survival of Testicular Pure Teratoma vs. Mixed Germ Cell Tumor Patients in Primary Tumor Specimens across All Stages

**DOI:** 10.3390/cancers15030694

**Published:** 2023-01-23

**Authors:** Cristina Cano Garcia, Francesco Barletta, Reha-Baris Incesu, Mattia Luca Piccinelli, Stefano Tappero, Andrea Panunzio, Zhe Tian, Fred Saad, Shahrokh F. Shariat, Alessandro Antonelli, Carlo Terrone, Ottavio De Cobelli, Markus Graefen, Derya Tilki, Alberto Briganti, Mike Wenzel, Severine Banek, Luis A. Kluth, Felix K. H. Chun, Pierre I. Karakiewicz

**Affiliations:** 1 Cancer Prognostics and Health Outcomes Unit, Division of Urology, University of Montréal Health Center, Montréal, QC H3A 2B4, Canada; 2 Goethe University Frankfurt, Department of Urology, University Hospital Frankfurt, 60590 Frankfurt, Germany; 3 Unit of Urology/Division of Oncology, Gianfranco Soldera Prostate Cancer Lab, IRCCS San Raffaele Scientific Institute, Vita-Salute San Raffaele University, 20132 Milan, Italy; 4 Martini-Klinik Prostate Cancer Center, University Hospital Hamburg-Eppendorf, 20246 Hamburg, Germany; 5 Department of Urology, IEO European Institute of Oncology, IRCCS, 20141 Milan, Italy; 6 Department of Urology, IRCCS Policlinico San Martino, 16132 Genova, Italy; 7 Department of Surgical and Diagnostic Integrated Sciences (DISC), University of Genova, 16132 Genova, Italy; 8 Department of Urology, University of Verona, Azienda Ospedaliera Universitaria Integrata di Verona, 37126 Verona, Italy; 9 Department of Urology, Comprehensive Cancer Center, Medical University of Vienna, 1090 Vienna, Austria; 10 Department of Urology, Weill Cornell Medical College, New York, NY 10065, USA; 11 Department of Urology, University of Texas Southwestern, Dallas, TX 75390, USA; 12 Hourani Center of Applied Scientific Research, Al-Ahliyya Amman University, Amman 19328, Jordan; 13 Department of Urology, University Hospital Hamburg-Eppendorf, 20246 Hamburg, Germany; 14 Department of Urology, Koc University Hospital, 34010 Istanbul, Turkey

**Keywords:** testicular cancer, pure teratoma, non-seminoma, survival analyses, SEER

## Abstract

**Simple Summary:**

Previous analyses from referral centers of testicular cancer investigated the prognostic impact of presence of teratoma components in advanced testicular primary tumor specimens and observed conflicting results. However, data investigating pure teratoma in primary tumor specimens is limited and the prognostic impact is uncertain. To address this void, we tested for overall survival differences and subsequently, differences in cancer-specific and other-cause mortality in pure teratoma vs. mixed germ cell tumor patients.

**Abstract:**

We aimed to test for survival differences between testicular pure teratoma vs. mixed germ cell tumor (GCT) patients in a stage-specific fashion. Pure teratoma and mixed GCT in primary tumor specimens were identified within the Surveillance, Epidemiology, and End Results database (2004–2019). Kaplan–Meier curves depicted five-year overall survival (OS) and subsequently, cumulative incidence plots depicted cancer-specific mortality (CSM) and other-cause mortality (OCM) in a stage-specific fashion. Multivariable competing risks regression (CRR) models were used. Of 9049 patients, 299 (3%) had pure teratoma. In stage I, II and III, five-year OS rates differed between pure teratoma and mixed GCT (stage I: 91.6 vs. 97.2%, *p* < 0.001; stage II: 100 vs. 95.9%, *p* < 0.001; stage III: 66.8 vs. 77.8%, *p* = 0.021). In stage I, survival differences originated from higher OCM (6.4 vs. 1.2%; *p* < 0.001). Conversely in stage III, survival differences originated from higher CSM (29.4 vs. 19.0%; *p* = 0.03). In multivariable CRR models, pure teratoma was associated with higher OCM in stage I (Hazard Ratio (HR): 4.83; *p* < 0.01). Conversely, in stage III, in multivariable CRR models, pure teratoma was associated with higher CSM (HR: 1.92; *p* = 0.04). In pure teratoma, survival disadvantage in stage I patients relates to OCM. Survival disadvantage in stage III pure teratoma originates from higher CSM.

## 1. Introduction

Treatment of testicular cancer is based on the stage and the distinctions between seminoma and non-seminoma [1]. However, within non-seminoma germ cell tumors (NSGCT), presence of potentially higher risk histological subtypes, such as presence of teratoma or even more so, presence of pure teratoma, received limited consideration in treatment and follow-up guidelines based on the rarity of this histological subtype. Specifically, based on the established chemo-refractory status of pure teratoma in primary specimens of retroperitoneal lymph node dissection (RPLND), subsequent chemotherapy is not recommended. Previous analyses derived from referral centers of testicular cancer investigated the prognostic impact of the presence of teratoma components in advanced testicular primary tumor specimens and observed conflicting results [2,3]. However, it is unknown to what extent the presence of pure teratoma in primary tumor specimens increases the risk of mortality relative to mixed GCT in metastatic, as well as in regional or localized stages. Moreover, it is unknown to what extent cancer-specific mortality (CSM) vs. other-cause mortality (OCM) contribute to overall survival (OS) across the three stages of testicular cancer in pure teratoma vs. mixed GCT. We addressed these information gaps. Specifically, we hypothesized that pure teratoma in primary tumor specimens confers a higher CSM risk than mixed GCT, across all stages. Conversely, we postulated that OCM rates are the same between pure teratoma and mixed GCT, also across all stages. To test these hypotheses, we relied on the Surveillance, Epidemiology and End Results (SEER) database (2004–2019). 

## 2. Materials and Methods

### 2.1. Patients

The SEER database approximates United States demographic composition and cancer incidence by collecting cancer incidence and survival data from population-based cancer registries. Specifically, United States death data are provided from the National Center of Health Statistics (NCHS) to the SEER database including the cause of death [4]. Within the SEER database from 2004 to 2019, we selected patients ≥18 years old, who underwent orchiectomy, with histologically confirmed non-seminoma testicular cancer (International Classification of Disease for Oncology [ICD-O] site codes C62.1, C62.9) of pure teratoma (ICD-O-3 histology code 9080/3) or mixed germ cell tumor histology (ICD-O-3 Codes 9085/3) in the primary tumor specimen. Teratoma patients with positive Alpha-Fetoprotein (AFP) or/and beta human chorionic gonadotropin (beta-hCG) tumor markers were excluded (n = 137). Additionally, autopsy only, as well as death certificate only, cases as reporting sources were excluded. Further exclusion criteria consisted of unknown clinical stage, missing follow up and survival data. OS, CSM (i.e., death from testicular cancer) and OCM (i.e., death not attributable to testicular cancer) were defined according to the SEER mortality code [4].

### 2.2. Statistical Analyses

All analyses stratified the population between pure teratoma vs. mixed GCT. Separate models were first applied to stage I patients, then to stage II and finally to stage III patients. Specifically, Kaplan–Meier analyses tested for OS differences between pure teratoma and mixed GCT. Cumulative incidence plots depicted CSM and OCM rates in pure teratoma vs. mixed GCT patients. Multivariable competing risks regression (CRR) models tested for independent predictor status of pure teratoma vs. mixed GCT in analyses addressing CSM after adjustment for OCM, as well as in analyses addressing OCM after adjustment for CSM. Co-variables consisted of age and RPLND status, as well as the International Germ Cell Cancer Collaborative Group (IGCCCG) risk group and presence of lung metastases [5] in stage III. In all statistical analyses, the R software environment for statistical computing and graphics (R version 4.1.3, R Foundation for Statical Computing, Vienna Austria) was used [6]. All tests were two-sided, with a significance level set at *p* < 0.05. Owing to the anonymously coded design of the SEER database, study-specific ethics approval was waived by the institutional review board.

## 3. Results

### 3.1. Descriptive Characteristics

Of 9049 study patients, 299 (3%) had pure teratoma vs. 8750 (97%) having mixed GCT. Median age was 29 and 28 years for pure teratoma and mixed GCT patients, respectively. Median follow-up was 70 months (interquartile range (IQR): 27–121). Pure teratoma differed from mixed GCT patients regarding a higher rate of performed RPLND (24 vs. 19%, *p* = 0.01). Conversely, pure teratoma patients exhibited a lower rate of IGCCCG intermediate risk group (2 vs. 22%, *p* = 0.002) and a lower rate of lung metastases than mixed GCT (24 vs. 43%; *p* = 0.01). No differences were observed regarding age at diagnosis, year of diagnosis, stage, as well as the IGCCCG good and poor risk groups (Table 1).

### 3.2. Overall Survival in the Overall Cohort of Pure Teratoma and Mixed Germ Cell Tumors

**Figure 1:**
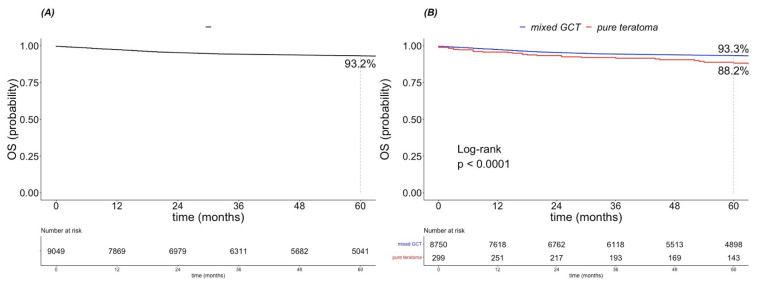
Kaplan–Meier estimates of (**A**) overall survival (OS) in 9183 patients and (**B**) OS after stratification according to pure teratoma vs. mixed germ cell tumor (GCT).

Five-year OS rates were 93.2% for all 9049 patients (Figure 1A). After stratification according to pure teratoma vs. mixed GCT, five-year OS rates were 88.2% in pure teratoma and 93.3% in mixed GCT patients (*p* < 0.001; Figure 1B). 

### 3.3. Overall Survival in Pure Teratoma versus Mixed Germ Cell Tumor Patients According to Stage

**Figure 2:**
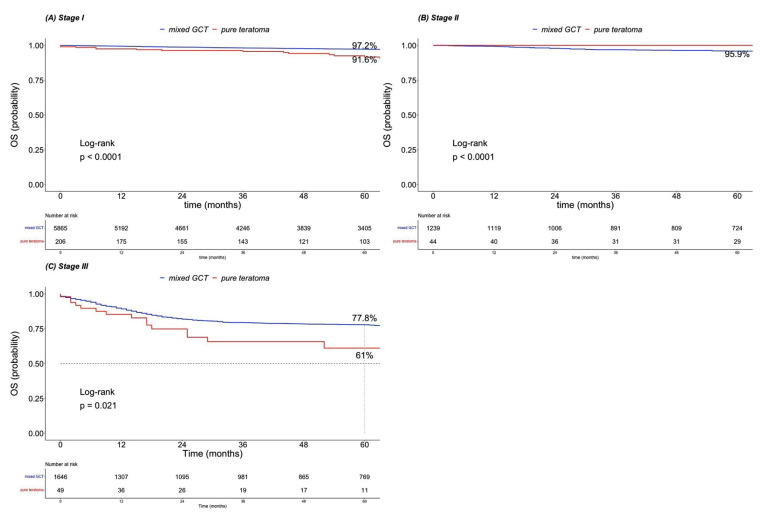
Kaplan–Meier estimates of overall survival (OS) in pure teratoma vs. mixed germ cell tumor (GCT) patients according to (**A**) stage I, (**B**) stage II and (**C**) stage III.

In stage I, five-year OS rates were 91.6 vs. 97.2% for, respectively, pure teratoma vs. mixed GCT (*p* < 0.001, Figure 2A). In stage II, five-year OS rates were 100 vs. 95.9% for, respectively, pure teratoma vs. mixed GCT (*p* < 0.001, Figure 2B). In stage III, five-year OS rates were 61.0 vs. 77.8% for, respectively, pure teratoma vs. mixed GCT (*p* = 0.021; Figure 2C).

### 3.4. Cancer-Specific Mortality in Pure Teratoma versus Mixed Germ Cell Tumor Patients According to Stage

**Figure 3:**
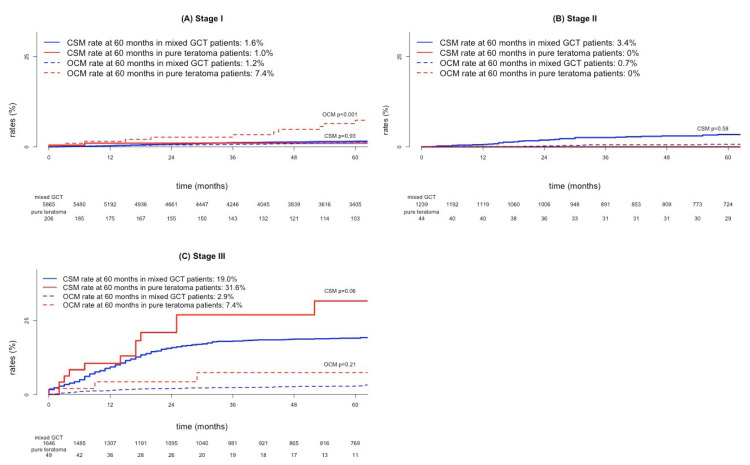
Cumulative incidence plots depicting cancer-specific mortality (CSM) and other-cause mortality (OCM) rates in patients with pure teratoma and mixed germ cell tumor (GCT) according to (**A**) stage I, (**B**) stage II and (**C**) stage III.

In stage I, five-year CSM rates were 1.0 vs. 1.6% for, respectively, pure teratoma vs. mixed GCT patients (*p* = 0.93; Figure 3A). In stage II, five-year CSM rates were 0 vs. 3.4% for, respectively, pure teratoma vs. mixed GCT (*p* = 0.58; Figure 3B). In stage III, five-year CSM rates were 31.6 vs. 19.0% for, respectively, pure teratoma vs. mixed GCT (*p* = 0.06; Figure 3C).

### 3.5. Other-Cause Mortality in Testicular Pure Teratoma Versus Mixed Germ Cell Tumor Patients According to Stage

In stage I, five-year OCM rates were 7.4 vs. 1.2% for, respectively, pure teratoma vs. mixed GCT patients (*p* < 0.01; Figure 3A). In stage II, five-year CSM rates were 0 vs. 0.7% for, respectively, pure teratoma vs. mixed GCT (Figure 3B). In stage III, five-year OCM rates were 7.4 vs. 2.9% for, respectively, pure teratoma vs. mixed GCT (*p* = 0.21; Figure 3C).

### 3.6. Multivariable Competing Risks Regression Models

In stage I, multivariable CRR models identified pure teratoma as an independent predictor of higher OCM relative to mixed GCT (Hazard Ratio [HR]: 4.83; 95% confidence interval [CI]: 2.83–8.24; *p* < 0.001). Conversely, pure teratoma did not predict higher CSM in stage I (HR: 0.98; 95% CI: 0.31–3.13, *p* = 0.98; Table 2).

In stage II, multivariable CRR models did not identify pure teratoma as an independent predictor in analyses addressing CSM, relative to mixed GCT (HR 2.01; 95% CI: 0.50–8.15, *p* = 0.33). 

In stage III, multivariable CRR models identified pure teratoma as an independent predictor of higher CSM relative to mixed GCT (HR: 1.92; 95% CI: 1.05–3.52, *p* = 0.04). Conversely, pure teratoma did not predict higher OCM in stage III (HR: 2.08; 95% CI: 0.67–6.48, *p* = 0.21; Table 2).

## 4. Discussion

It is unknown to what extent the presence of pure teratoma in primary tumor specimens increases the risk of mortality relative to mixed GCT in metastatic, as well as in regional or localized stage testicular cancer. Moreover, it is unknown to what extent CSM vs. OCM contribute to OS across the three stages of testicular cancer in pure teratoma vs. mixed GCT patients. We addressed these information gaps and hypothesized that higher CSM applies to pure teratoma vs. mixed GCT, across all stages. Conversely, we postulated that OCM rates are the same between pure teratoma vs. mixed GCT patients, across all stages. We tested these hypotheses within the 2004–2019 SEER database and made several important observations.

First, teratoma is indeed a rare entity. The number of teratoma patients identified within the current study (n = 299, 3%) is very low compared to the number of patients with mixed GCT (n = 8750, 97%). Although data investigating pure teratoma are limited, the proportion of patients harboring pure teratoma in primary tumor specimens of the current study is in agreement with previous studies where this proportion ranged from 2 to 6% [7,8,9,10,11,12,13]. The very close agreement between pure teratoma rate in the current study (3%) and the range of pure teratoma rates in previous studies (2–6%) validates the concept of pure teratoma definition within the SEER database and in consequence, within the current study.

Second, we observed important OS differences between pure teratoma vs. mixed GCT. At five years of follow-up, OS rates were 88.2 vs. 93.3% for, respectively, teratoma and mixed GCT patients (*p* < 0.001). Further stratification according to stage revealed important OS differences between pure teratoma vs. mixed GCT patients in stage I (91.6 and 97.2%; *p* < 0.001) and in stage II (100 vs. 95.9%, *p* < 0.001), as well as in stage III (66.8 and 77.8%; *p* < 0.021). The current observations regarding OS in stage III are in agreement with Funt et al. where metastatic testicular cancer patients with presence of teratoma vs. those without presence of teratoma were compared [2]. Conversely, Taza et al. reported no OS differences between testicular cancer with vs. without presence of teratoma [3]. To the best of our knowledge, a design that perfectly replicates the current design (pure teratoma vs. mixed GCT in primary tumor specimens) has not been completed. In consequence, the current study’s findings cannot be directly compared to any previous works.

Third, we recorded important findings testing for CSM differences between pure teratoma and mixed GCT. Specifically, in stage III, we recorded higher CSM in pure teratoma vs. mixed GCT (31.6 vs. 19.0%, *p* = 0.06; multivariable HR 1.92, *p* = 0.04). In stage I and stage II, no CSM differences were recorded between pure teratoma vs. mixed GCT (stage I 1.0 vs. 1.6%, *p* = 0.93; stage II 0 vs. 3.4%, *p* = 0.58). The observation addressing higher CSM in pure teratoma vs. mixed GCT in stage III cannot be directly compared to previous studies since no previous study distinguished between CSM vs. OCM in either stage III, II or I patients. However, our findings regarding higher CSM in pure teratoma vs. mixed GCT in stage III substantiate the observation of Funt et al., who reported higher CSM in metastatic patients with presence of teratoma vs. absence of teratoma [2]. Specifically, our findings add to the existing evidence and illustrate that OS in stage III pure teratoma patients originated from higher CSM, but is unrelated to OCM differences. 

Fourth, we also recorded differences in OCM between pure teratoma and mixed GCT patients. Specifically, OCM was higher in stage I pure teratoma than mixed GCT (7.4 vs. 1.2%, *p* < 0.001; multivariable HR 4.83, *p* < 0.001). No statistically significant OCM differences between pure teratoma vs. mixed GCT were recorded in stage II (0 vs. 0.7%) and stage III (7.4 vs. 2.9%; *p* = 0.21). To the best of our knowledge, we are the first to address differences in OCM rates between pure teratoma vs. mixed GCT across all stages. In consequence, our results cannot be directly compared to existing results. However, abundant data exist regarding higher rates of comorbidities after exposure to systemic therapy, which may have been administered at a higher rate to pure teratoma patients than to mixed GCT counterparts [14,15,16]. Conversely, for stage I, the existing evidence about late toxicity and mortality after a single dose of bleomycin, etoposide, cisplatin (BEP) does not indicate a disadvantage to PEB-exposed patients [17,18,19,20,21,22,23]. This said, the nature of the SEER database does not allow to examine recurrence and progression rates that would result in stage reclassification towards higher stages where additional systemic therapy or RPLND, or both were required. Based on existing data, such salvage regimens result in favorable cancer-specific survival rates [23,24]. However, they may leave a mark on OCM from potential untoward effects that may be captured in the form of a higher OCM signal. This hypothesis clearly required validation within other databases.

Taken together, the current study validates the rarity of pure teratoma. Moreover, our analysis demonstrated differences in OS between pure teratoma and mixed GCT patients in stage I, stage II and stage III. In stage I, pure teratoma patients whose OS was lower than that recorded in their mixed GCT counterparts; we also recorded higher OCM. The precise cause of this observation cannot be explained with SEER data alone, but suggests the need for further analyses aimed at identifying the underlying etiology of this observation or alternatively refuting the association that we observed. Conversely, in stage III pure teratoma patients whose OS was lower than that recorded in their mixed GCT counterparts, we also recorded higher CSM. These observations indicate the unfavorable effect on cancer control outcomes in stage III pure teratoma vs. mixed GCT. Similar observations and interpretations were made by Funt et al. within a somewhat different study design, where presence of teratoma was compared to absence of teratoma in metastatic NSGCT [2]. Moreover, in stage II patients, pure teratoma patients exhibited higher OS than their mixed GCT counterparts (100 vs. 95.9%; *p* < 0.001). However, no difference in CSM and OCM between stage II pure teratoma and mixed GCT patients were observed. Cary et al. reported, in a series of 14 stage II pure teratoma and chemo naïve patients undergoing RPLND, good clinical outcomes [25]. Conversely, no comparison between pure teratoma and mixed GCT patients was performed in the study by Cary et al. Finally, the findings of the current study emphasize the importance of CSM and OCM quantification in both stage I and stage III patients. These observations are of an explorative nature. Further studies designed to validate or reject our findings are needed. 

Despite its novelty, the current study has several limitations. The first and foremost limitation consists of patient origin. Specifically, our findings are applicable to individuals who are identified within the SEER database. In consequence, the observations made within the current study are not generalizable to individuals from outside the United States or even patients who are not comparable to those included in the SEER database. Therefore, institutional series or multi-institutional series reflecting cancer control outcomes of such individuals should be used if available. Moreover, the results of the current study are not comparable to high volume hospitals that were previously identified to be associated with increased overall survival of testicular cancer patients [26]. No information on hospital volume is given in the SEER database. In consequence, no consideration or adjustment for hospital volume was possible. Second, histological information other than from primary tumor specimen is not available. Moreover, no information about residual retroperitoneal lymph node lesions after chemotherapy is available. Third, our analyses relied on a limited number of observations. Especially in stage II, the comparison between pure teratoma and mixed GCT relied on limited events. This sample size limitation represented an important factor even within the current, very large-scale analysis. In consequence, it is unlikely that smaller-scale databases, except for the National Cancer Database (NCDB), will provide more robust results. Fourth, the median follow-up of 6 years (70 months) represents another limitation of the current study. Ideally, a longer follow-up would be of value, especially for analyses regarding stage I and stage II. Fifth, no information about centralized pathological review, as well as on immunohistochemistry, was available within the SEER database. Sixth, SEER lacks specific histological information about the composition of mixed GCT and the percentages of various components within the primary. In consequence, the same study design as applied by Funt et al. and Taza et al. cannot be applied directly to the SEER database [2,3]. Seventh, the currently used SEER database does not provide information on systemic therapy. Moreover, the SEER database does not provide the granularity to identify specific systemic therapy regimens and does not provide information on the number of cycles and duration of its administration. Finally, no information on rates of growing teratoma syndrome in pure teratoma patients was available. 

## 5. Conclusions

In testicular pure teratoma, the survival disadvantage in stage I patients was previously unknown and relates to OCM. The survival disadvantage in stage III pure teratoma originates from higher CSM that was also previously unknown.

## Figures and Tables

**Table 1:** Baseline characteristics of 9049 patients with pure teratoma and mixed germ cell tumor (GCT) within the Surveillance, Epidemiology and End Results (SEER) database (2004–2019).

Characteristic	Pure Teratoma ^1^ n = 299 (3%)	Mixed GCT ^1^ n = 8750 (97%)	*p*-Value ^2^
Age at diagnosis	29 (23, 36)	28 (24, 35)	0.8
Year of diagnosis			0.07
2004–2011	113 (38%)	3775 (43%)	
2012–2019	186 (62%)	4975 (57%)	
Stage			0.6
I	206 (69%)	5865 (67%)	
II	44 (15%)	1239 (14%)	
III	49 (16%)	1646 (19%)	
RPLND performed	73 (24%)	1619 (19%)	0.01
IGCCCG risk group for stage III (n = 1695)	n = 49	n = 1646	
Good prognosis	10 (20%)	271 (16%)	0.6
Intermediate prognosis	1 (2%)	369 (22%)	0.002
Poor prognosis	20 (41%)	639 (39%)	0.9
Unknown	18 (37%)	367 (22%)	0.003
Lung metastases	12 (24%)	713 (43%)	0.01

^1^ Median (IQR); n (%). ^2^ Wilcoxon rank sum test; Pearson’s Chi-square test; Fisher’s exact test. Abbreviations: RPLND = Retroperitoneal Lymph Node Dissection, IGCCCG = International Germ Cell Cancer Collaborative Group.

**Table 2:** Multivariable competing-risk regression models predicting cancer-specific mortality (CSM) and other-cause mortality (OCM) in patients with pure teratoma or mixed germ cell tumor (GCT) stages I, II (adjusted for age at diagnosis, retroperitoneal lymph node dissection) and stage III (adjusted for age at diagnosis, retroperitoneal lymph node dissection, International Germ Cell Cancer Collaborative Group risk group, presence of lung metastases).

		CSM		OCM	
		HR (95% CI)	*p*-Value	HR (95% CI)	*p*-Value
Stage I					
Histology	mixed GCT	Reference	-	Reference	-
	pure teratoma	0.98 (0.31–3.13)	0.98	4.83 (2.83–8.24)	<0.001
Stage II					
Histology	mixed GCT	Reference	-	Reference	-
	pure teratoma	2.01 (0.50–8.15)	0.33	-	-
Stage III					
Histology	mixed GCT	Reference	-	Reference	-
	pure teratoma	1.92 (1.05–3.52)	0.04	2.08 (0.67–6.48)	0.21

## Data Availability

All data generated for this analysis were from the Surveillance, Epidemiology and End Results (SEER) database. The code for the analyses will be made available upon request.
